# A blended knowledge translation initiative to improve colorectal cancer staging [ISRCTN56824239]

**DOI:** 10.1186/1472-6963-6-4

**Published:** 2006-01-16

**Authors:** Frances C Wright, Calvin HL Law, Linda D Last, Neil Klar, David P Ryan, Andrew J Smith

**Affiliations:** 1 Sunnybrook & Women's College Health Sciences Centre, University of Toronto, T-Wing, 2075 Bayview Ave., Toronto, ON, Canada, M4N 3M5; 2 Department of Epidemiology and Biostatistics, University of Western Ontario, London, ON, Canada N6A 5C1

## Abstract

**Background:**

A significant gap has been documented between best practice and the actual practice of surgery. Our group identified that colorectal cancer staging in Ontario was suboptimal and subsequently developed a knowledge translation strategy using the principles of social marketing and the influence of expert and local opinion leaders for colorectal cancer.

**Methods/Design:**

Opinion leaders were identified using the Hiss methodology. Hospitals in Ontario were cluster-randomized to one of two intervention arms. Both groups were exposed to a formal continuing medical education session given by the *expert opinion leader for colorectal cancer*. In the treatment group the local Opinion Leader for colorectal cancer was detailed by the *expert opinion leader for colorectal cancer *and received a toolkit. Forty-two centres agreed to have the *expert opinion leader for colorectal cancer *come and give a formal continuing medical education session that lasted between 50 minutes and 4 hours. No centres refused the intervention. These sessions were generally well attended by most surgeons, pathologists and other health care professionals at each centre. In addition all but one of the local *opinion leaders for colorectal cancer *met with the *expert opinion leader for colorectal cancer *for the academic detailing session that lasted between 15 and 30 minutes.

**Discussion:**

We have enacted a unique study that has attempted to induce practice change among surgeons and pathologists using an adapted social marketing model that utilized the influence of both expert and local opinion leaders for colorectal cancer in a large geographic area with diverse practice settings.

## Background

A significant difference exists between what is known to represent best practice and the actual practice of medicine [1]. Such gaps have been repeatedly documented in surgical practice [2-9]. Unfortunately, traditional continuing professional development (CPD) has not been able to bridge the gap between research and evidence-based guidelines and clinical practice [10-13] and consequently, many have advocated for an improved approach [14,15]. Davis has advocated for 'knowledge translation', as a way of closing the gap between evidence and practice [1]. Such an approach focuses on situated learning within the actual practice of healthcare, includes all participants involved in healthcare practices and attempts to identify and overcome local barriers that prevent practice change [1].

Our group has focused on identifying and closing a gap in colorectal cancer (CRC) staging in Ontario. Accurate staging of CRC is critical when determining appropriate treatment for patients and when it is poorly done it has a negative impact on patient survival [16,17]. Optimal CRC staging involves clinician knowledge of the minimum number of lymph nodes (LN) to be assessed, requires that a surgeon perform an appropriate mesenteric resection and a pathologist identify and assess the LNs adequately [18]. In Ontario, where 73% of patients with Stage II CRC were shown to be inadequately staged [7], the challenge of influencing the practice of over 1000 surgeons and pathologists in multiple institutions is significant. We examined the effectiveness of a multi-pronged strategy attempting to improve CRC staging in this large geographic area.

We embarked upon a knowledge translation study in which we adapted the principles of social marketing to influence physician behaviour in the context of a practice element that has both knowledge and technical aspects [19]. In contrast, Soumerai initially described social marketing in the context of changing physician prescribing practices [19]. The elements of the social marketing approach include defining clear educational objectives, focusing programs on specific categories of physicians and opinion leaders, establishing 'messenger' credibility, stimulating physician participation in educational interactions, using concise educational materials and providing positive reinforcement when trying to change physician behaviour. A recent Cochrane Review has suggested that social marketing can change physician behaviour [20].

Embedded in the social marketing model we adapted, was the identification (using Hiss methodology) and utilization of both local and expert opinion leaders' (OL) influence [21]. According to the Hiss construct, local OLs are physicians who 1) encourage learning and enjoy sharing their knowledge; 2) are clinical experts and always seem up-to-date and 3) treat others as equals [21,22]. Opinion leaders who are influential in inciting change at their local health care institution are considered local to peer Ols [23]. OLs who are considered influential on a provincial or national level are considered 'expert' OLs and their support for evidence is sufficient endorsement to consider adoption [24]. Such expert OLs are often consultants or work out of academic centres [25]. Among surgeons, Young has identified that the concept of the OL, especially on a provincial or national level, is supported and that OLs, are considered influential [26].

Little has been written about how large-scale social marketing that utilizes the influence of local and expert opinion leaders has been performed among specialist physicians. The Cochrane Collaboration suggests that further research is required to determine in what context social marketing and OLs are most likely to influence the practice of their peers [20,27]. In this paper we describe a knowledge translation intervention that used the principles of social marketing and that utilized the influence of local and expert Ols [19,28].

## Methods

### Study design and study population

**Figure 1:**
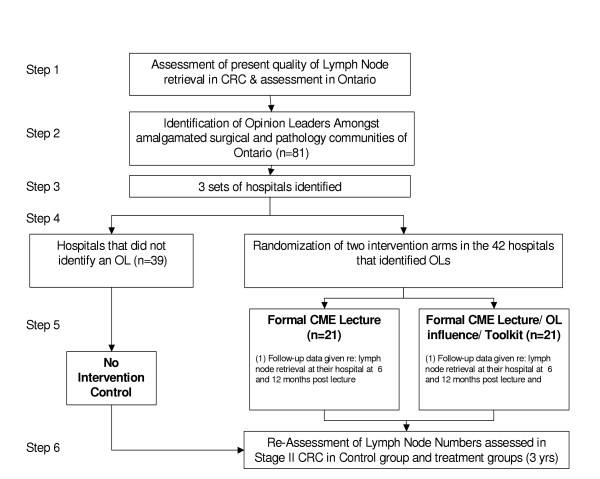
Randomization Algorithm for Ontario Hospitals

This trial compared formal CME alone and formal CME plus the influence of a local opinion leader plus a toolkit on changing surgeon and pathologist behaviour in the context of LN assessment in CRC staging. It was a cluster-randomized trial with the hospital as the unit of random assignment [29]. The particular design was utilized to minimize the contamination of education materials between intervention arms and enabled measurement of the rate of change in each region (Fig. 1) [29]. Most randomized trials do not have outcome data for non-participants. This limits their ability to generalize study results or to determine the impact of Hawthorne effects [30,31]. A strength of our study is that the number of LN's assessed will be available for all patients in Ontario diagnosed with CRC. Consequently we will also be comparing LN assessment of patients from the study hospitals to those of patients whose LN's were assessed at hospitals which did not participate in our trial.

### Sample size and power calculation

Between 1997 and 2000, 27% of patients had an appropriate number of LNs (minimum 12) examined in their surgical specimens in Ontario [7]. As part of this initiative we enrolled 42 hospitals in Ontario. Twenty-one hospitals were randomized to a formal continuing medical education lecture and the other 21 were randomized to the lecture/OL influence/toolkit arm. The remaining hospitals that did not identify an OL did not participate in the study. This sample size is predicted to have 80% power to detect a difference between the two randomized treatment arms of 27% for CME alone and 54% for CME plus the influence of a local opinion leaders plus toolkit in the proportion of patients having at least 12 nodes examined assuming an average of 3 patients per hospital at the six month assessment and using a two-tailed 5% type I error rate and an intracluster correlation coefficient of 0.1. Note that the assumption that 27% of patients seen at CME alone hospitals will have at least 12 nodes assessed is equivalent to what was seen, overall, by Wright et al. as was the degree of intracluster correlation [7]. A two fold difference between the randomized arms was selected as being clinically relevant and equivalent to the difference found between academic and nonacademic hospitals by Wright et al [7]. The sample size was determined using the approach described by Donner and Klar [29]. Greater power is anticipated to be achieved as the primary analysis is based on the number of nodes examined per patient using the generalized estimating equations extension of Poisson regression to account for between hospital variation in node counts [29].

### Opinion leader identification

A two-page survey instrument was created based on the OL identification methodology developed by Hiss (Additional file: 1) [32]. The wording that was used to describe the three OL characteristics (knowledgeable practitioners, educators, and caring professionals who exhibit a high level of humanist concern) was minimally changed from the original Hiss survey instrument [33]. We then identified OLs with special expertise in CRC using a technique utilized by other investigators who also identified OLs with special expertise [34-39]. Importantly, in Soumerai's, Gifford's, Lomas' and Guadagnoli's study [34], the OLs (with special expertise) were part of a multi-modal approach that resulted in an improvement in patient care.

### Sample

The OL survey was mailed to 1243 general surgeons and pathologists in Ontario with a stamped addressed envelope (SAE) for their reply. Physicians were identified using the Canadian Medical Directory™ (2001) using the criteria "General Surgeons and Pathologists *with and without *hospital affiliation". The second mail contact included the survey, SAE and an incentive to complete the survey (draw for a Sony Clie^©^™). An electronic mail reminder to fill in the survey, including incentive information, was sent out to 366 physicians (e-mail addresses from the Canadian Medical Directory™). A subset of physicians who had not replied after two mailings had their offices contacted by telephone and if they agreed, a survey was faxed to their office or completed over the phone. The third mail contact again included a SAE, survey, and the same incentive. The final mail contact included the survey, SAE, the same incentive and a flyer with the TNM Staging for colorectal cancer. All forms of contact were coded so that it could be identified which contact had initiated the survey response.

Physicians were excluded if their practice did not include any colorectal cancer surgery and if they were practicing in another surgical sub-specialty i.e. cardiac, thoracic, vascular surgery or if they were not currently practicing in Ontario, retired or deceased. Physicians whose surveys were returned with an incorrect address were also excluded. Efforts were made to find the correct address including using the College of Physicians and Surgeons of Ontario (CPSO) website and the Royal College of Physicians and Surgeons of Canada (RCPSC) website. "Refusals" were defined as physicians who sent back the surveys not completed or partially completed.

### Hospital amalgamation

In 1997–2000, we identified 99 hospitals that performed CRC surgery in Ontario. By the time we identified OL in Ontario, a number of hospitals had been amalgamated at the request of the provincial government. Hence at the time of our study, 81 hospitals were performing CRC surgery.

### Identifying an opinion leader for colorectal cancer

In our study, physicians were defined as an *OL for CRC *if they were recognized at least once in all three OL categories and additionally were identified as a person whose advice was valued on CRC using this algorithm. Forty-two hospitals identified an *OL for CRC *and 39 centres did not, one of which was an academic centre [22]. The 42 hospitals that identified an OL participated in the education intervention and the 39 that did not were not exposed to an intervention. Importantly, our lack of OL identification in a number of centres is not unique, Young (2003) did not identify local surgical OLs at each hospital in her study of Australian surgeons [26].

### Identifying the characteristics of an "*Expert OL for CRC*"

The *expert OL *was a highly regarded surgeon who was identified (multiple times) as an *OL for CRC*, worked at an academic centre, is recognized in the surgical community as having expertise in CRC on the basis of multiple formal Continuing Medical Education presentations and publications regarding CRC and for treating a high volume of patients with CRC.

### Randomization

Hospitals that identified OL's were cluster-randomized to one of two arms. The intervention arm included a Formal CME (continuing medical education) lecture plus the influence of the Opinion Leader plus a Toolkit. The control arm was the Formal CME session alone. The cluster randomization design was utilized to minimize the contamination of education materials between intervention arms and enabled measurement of the rate of change in each region (Fig. 1) [29]. In addition, hospitals were stratified, prior to random assignment, based on two characteristics, their academic status and their yearly volume of CRC cases (low < 20, high ≥ 20). Our previous work demonstrated that a hospital's designation as an academic or non-academic institution was an important predictor of the number of LNs assessed in CRC [7]. We defined academic centers as those hospitals in which both pathology and surgery residents have regular rotations. The remaining hospitals that did not identify *OL for CRC *and did not participate in the education sessions constituted the control arm (see Fig 1).

### Intervention

All randomized hospitals (n = 42) participated in the formal educational sessions led by the same *expert OL for CRC*. The sessions emphasized the importance of adequate LN assessment in CRC and consisted of a standardized PowerPoint^® ^lecture which had previously been tested in a single institution study and an interactive question and answer session [18]. The presentation also included a 'scatter gram' of the median LN counts collected for patients with Stage II CRC (1997–2000) for each hospital in the province. If requested by the institution or an individual surgeon/pathologist, the median LN count for that particular institution was revealed at a later date. Participants in the study were not made aware that they were part of a randomized controlled trial and that CRC LN counts would be reassessed after the formal education sessions and academic detailing had been completed.

### Opinion leader arm

The *expert OL for CRC *met with all but one of the locally identified OL and discussed the importance of adequate LN assessment, local barriers toward improving LN assessment and possible solutions were discussed (academic detailing). A toolkit was provided at the time of the detailing for the OL which consisted of a binder that had a cover letter, a print-out of the PowerPoint^® ^slide presentation, a pathology template for CRC, guidelines for what should be included in a pathology report for CRC, 3 copies of a poster with a picture of a colon with a number '12' watermarked over it and 3 pocket cards that reminded the OL what an optimal pathology report for CRC would look like, the AJCC staging and a reminder of the minimum number of LN to assess in a CRC specimen. Further printed information was given to some OLs either during the sessions or after if requested. E-mail and telephone follow-up also occurred with some OLs.

#### Follow-up

A follow-up package was sent 6 months following the presentation (regular mail) to the CME plus OL plus toolkit site only. The package included a cover letter from the *expert OL for CRC *thanking the local OL for their participation in the process and opening the door to further discussion if requested, a peer-reviewed paper on LNs clearing solution and more of the same pocket cards [40].

Ethical Review was obtained from the Sunnybrook and Women's College Health Sciences Centre Research Ethics Review Board to complete this trial.

### Preliminary results

#### Participation

All centres (n = 42) agreed to have the *expert OL for CRC *come and give the formal CME session. Although the hospitals were enthusiastic about hosting the sessions, the arrangements to coordinate the sessions were time consuming. Ninety-percent (38/42) of these sessions took place over a four-month time period beginning in January 5^th ^2004 and all of the sessions were completed by June 17^th ^2004 (5.5 months). Twenty-four of the sessions were given in the context of hospital rounds and 18 were formal dinners at local restaurants. In general, the dinner presentations were organized for smaller community hospitals. The sessions varied in length between 50 minutes to 4 hours. Food was paid for by the research group in 20/42 centres. Attendance was generally good with most surgeons, pathologists, and pathology assistants (if the position existed at the hospital) attending at each centre. In addition, medical oncologists, family physicians, residents, fellows and nurses also attended some of the sessions. All but one of the opinion leaders in the academic detailing arm agreed to meet personally with the *expert OL for CRC *(96%) on an individual basis. These sessions lasted between 15–30 minutes. The local OL who did not meet personally with the expert OL for CRC communicated with the *expert OL *on the telephone and via electronic mail.

#### Feedback

Hospitals and OLs relayed their enthusiasm for the project in a number of different ways. Two centres performed audits on the local LN counts prior to the CME session by the *expert OL for CRC*. After the session was completed, five centres sent correspondence relaying the positive experience of the session, four centres indicated that they were planning to initiate a multi-disciplinary gastro-intestinal tumour conference and four centres requested further information (particularly with regards to their own LNs counts). Interestingly, two centres that were not visited as part of the study also contacted our group requesting information on how to improve their LN counts in CRC.

## Discussion

This study is unique in attempting to incite practice change amongst surgeons and pathologists by using an adapted social marketing model. The intervention utilized the influence of Hiss-criteria OLs for CRC in a large geographic area with diverse practice settings (Table 1) [21,38,39,41-43]. Importantly, surgeons have indicated in a number of studies that OLs could be influential on their practice patterns especially at a national level [22,26] (Simunovic, personal communication 2004). Social marketing has been successfully used to change physician behaviour primarily with respect to physician prescribing practices [44-46]. Our study is unique in that we have adopted this model to change aspects of physician behaviour that are related to a process that has both knowledge and technical elements.

We have utilized both the influence of an *expert OL for CRC *and a local or peer *OL for CRC *both of whom have different roles during a change intervention. Locock suggests that expert OLs are important in the initial stages of an education intervention when evidence needs to be endorsed and translated into a form that is acceptable to local practitioners [47]. The expert OL is considered to be a "higher authority" who is able to explain and evaluate the evidence [48]. In contrast, local or peer OLs are important in the later phases of implementation during which time they role model new behaviour and give colleagues confidence to initiate change [49].

In the present intervention, the *expert OL for CRC *personally went to each of the hospitals (n = 42) to present the CME session (endorse the evidence) and detail the local OLs. We feel that the presentation by the *expert OL for CRC *at each local site was an important aspect of the intervention. It has been demonstrated that when physicians consider adopting an innovation that the meaning of the new information has to be discussed among the local physicians (social construction of knowledge) before it can be enacted [50, 51, 52, 53, 54]. We postulate the presence of the *expert OL for CRC *when endorsing the new information initiated this discussion for each group and the local OL and will facilitate the process of behaviour change.

This multi-pronged intervention required a substantial time and financial commitment to enact. One full-time research assistant was employed to co-ordinate the formal CME sessions, academic detailing and follow-up packages. The *expert OL for CRC *also dedicated 25% of his clinical time for four months to complete the intervention. Clearly this sort of intervention is resource intense and if it is to become a standard approach to changing physician behaviour then substantial support would be required.

There are a number of reasons why this study may not be successful in achieving its endpoint of improved CRC staging. First, during our OL identification process we may not have included a number of new general surgery graduates in our survey mail out and hence, some *OL for CRC *may not have been identified. Second, our intervention predominantly addressed a knowledge deficit and although we attempted to incite discussion and start the process of the social construction of knowledge, if local barriers were too great (i.e. pathology resource issues) then improving CRC staging may be an insurmountable barrier [55].

However, if the intervention is successful then we may not be able to clearly determine if *our *intervention alone was the catalyst. A number of other continuing medical education events have occurred in the province (including our own oncology rounds) that focused on accurate staging in CRC. In addition, the provincial Cancer Care Organization has also been recently focusing on improving the care of patients with cancer [56]. To address this issue, we will be collecting LN counts for a number of years (2002, 2003) before the intervention was initiated in 2004 as well as after the intervention was started. Our timeline involves data collection between September and December 2005, and analysis is to start in January 2006. In addition, we plan on conducting qualitative interviews with participants to determine whether our intervention incited any physician behaviour change.

Davis has suggested that further research is required to understand how to best facilitate the rapid uptake of evidenced-based knowledge [1]. We have described a knowledge translation initiative that we have implemented across Ontario with the aim of improving colorectal cancer staging. We have used a social marketing approach that included the influence of both peer and expert opinion leaders in an attempt to locally construct knowledge to enable physician behaviour change [19].

## Competing interests

The author(s) declare that they have no competing interests.

## Authors' contributions

FCW helped develop the study design, assisted with statistical analysis and drafted the manuscript. CHLL helped develop the study design, performed statistical analysis and helped draft the manuscript. LDL helped develop the study design, collected and collated the data and helped draft the manuscript. NK helped develop the study design and formulated the randomization and helped draft the manuscript. DPR helped develop the study design and helped draft the manuscript. AJS conceived and helped develop the study design, was the investigator who actually contacted and detailed the Opinion Leaders, helped draft the manuscript and helped with statistical analysis. All authors read and approved the final manuscript.

## Pre-publication history

The pre-publication history for this paper can be accessed here:



## Supplementary Material

Additional file 1 . Click here for file

## Figures and Tables

**Table 1:** A social marketing approach to changing physician behaviour [19]

**Steps in the social marketing approach**	**Description of intervention**
1. Conducting interviews to investigate baseline knowledge	A needs assessment was previously completed to assess how many LNs were being assessed in colorectal cancer staging [7]
2. Focusing programs on specific categories of physicians as well as on their opinion leaders	General Surgeons and Pathologists were targeted. Opinion leaders were identified and recruited [22]
3. Defining clear educational and behavioural objectives	LNs staging was emphasized during the discussion, including the target number of nodes to assess (12)
4. Establishing credibility through respected organizational identity, referencing authoritative literature and presenting controversies	The expert OL came from a respected organization. The formal CME discussion presented an overview of the literature
5. Stimulating physician participation	A question and answer session occurred after the formal CME session. This session often lasted long than the 'official' talk.
6. Using concise educational materials	The pocket cards and posters emphasized the importance of 12 LNs being assessed
7. Highlighting and repeating essential messages	The concept of 12 LNs was repeated in the talk, in the educational materials and in the reminder materials sent to the opinion leaders
8. Providing positive reinforcement of the improved practices in follow-up visits	Ongoing process. Reinforcement will be sent by mail.
