# Supplementary material for: A blended knowledge translation initiative to improve colorectal cancer staging [ISRCTN56824239]
Source: BMC Health Serv Res. 2006 Jan 16;6:4. doi: 10.1186/1472-6963-6-4 (PMC1395360; doi:10.1186/1472-6963-6-4)
Supplement: Additional file 1 — . [file 1472-6963-6-4-S1.pdf]

Appendix I: Hiss Opinion Leader Identification Survey

***(Please Print)***

Please read the following items and think about the physicians in your community. If possible, please list as many as two physicians in your community who best fit each description.

***NOTE: You may include the same physician in more than one section and you may include your own name in any section that you feel is appropriate.***

**I. PHYSICIANS AS EDUCATORS**

These physicians convey information in ways that lead to learning. They express themselves clearly and to the point. They provide practical information first and then an explanation or rationale if time allows. They take the time to answer you completely and do not leave you feeling that they were too busy to answer your inquiry. They enjoy and are willing to share any knowledge that they have.

Name: ___________________________Name:_____________________________

**II. PHYSICIANS AS KNOWLEDGEABLE PRACTITIONERS.**

These are physicians who like to teach. They seem always up-to-date; demonstrate a command of recent medical knowledge and a high level of clinical expertise.

Name: ___________________________Name:_____________________________

**III. PHYSICIANS AS CARING PROFESSIONALS**

These are physicians whom we might call real humanists. They treat people as equals; they listen, communicate well and never talk down to others even when helping them.

Name: __________________________Name:_____________________________

**IV. GENERAL SURGEONS WHOSE ADVICE YOU VALUE ON COLORECTAL CANCER**

Name: ___________________________Name:_____________________________

V. **PATHOLOGISTS WHOSE ADVICE YOU VALUE ON COLORECTAL CANCER**

Name: ___________________________ Name:_________________________________

**VI. DESIGNATED LEADERS IN YOUR COMMUNITY**

E.g. - General Surgery Division Head and Head of Pathology.

**SURGERY:** _______________________________________________________

**PATHOLOGY:** ____________________________________________________

**Please go to next page➔**

**Would you mind telling us a little about yourself?**

Gender: Female ☐ Male ☐ Age: ______ Years in Practice: _____

Practice Location: Rural ☐ Urban ☐ Remote ☐

Nature of your Practice (e.g. General Surgery or Pathology, subspecialty surgery or pathology):
______________________________________________________________________

Estimated percentage of your clinical volume that relates to colorectal cancer: _____

How important are the following resources when you need medical information **quickly**?

| | **Level of importance** | | | |
|---|---|---|---|---|
| Scientific and Professional Journals | Very ☐ | Moderately ☐ | Somewhat ☐ | Not ☐ |
| Online databases such as Medline | Very ☐ | Moderately ☐ | Somewhat ☐ | Not ☐ |
| Other internet resources or email | Very ☐ | Moderately ☐ | Somewhat ☐ | Not ☐ |
| Hospital physicians | Very ☐ | Moderately ☐ | Somewhat ☐ | Not ☐ |
| University faculty | Very ☐ | Moderately ☐ | Somewhat ☐ | Not ☐ |
| Informal communication with a specialist | Very ☐ | Moderately ☐ | Somewhat ☐ | Not ☐ |
| Formal CE | Very ☐ | Moderately ☐ | Somewhat ☐ | Not ☐ |
| The opinion leader physicians in your community | Very ☐ | Moderately ☐ | Somewhat ☐ | Not ☐ |
| Informal communication with other colleagues | Very ☐ | Moderately ☐ | Somewhat ☐ | Not ☐ |

Think about the physicians whom you approach for medical information or advice and let us know how you chose them (you can select more than one reason).

_____ Knew them in medical school
_____ Heard of them through talking with other colleagues
_____ Heard of them through interaction with hospital departments
_____ Met them at professional medical meetings
_____ Met them at informal social gatherings
_____ Work in the same building

Any other reasons? ________________________________________________________________
______________________________________________________________________________

When you have completed the materials would you kindly put them in the enclosed self-addressed envelope and drop it in the mail?

Once again, thank you very much for your help.
